# The Effects of Working Memory and Probability Format on Bayesian Reasoning

**DOI:** 10.3389/fpsyg.2020.00863

**Published:** 2020-05-12

**Authors:** Lin Yin, Zifu Shi, Zixiang Liao, Ting Tang, Yuntian Xie, Shun Peng

**Affiliations:** ^1^Cognition and Human Behavior Key Laboratory of Hunan Province, School of Educational Science, Hunan Normal University, Changsha, China; ^2^School of Psychology, Central China Normal University, Wuhan, China

**Keywords:** working memory, probability format, Bayesian reasoning, dual-process, cognitive resource

## Abstract

Bayesian reasoning is common and critical in everyday life while the performance on Bayesian reasoning is rather poor. Previous studies showed that people could enhance their performance by applying cognitive resources under the natural frequency format condition. Working memory is one of the crucial cognitive resources in the reasoning process. However, the role of working memory on Bayesian reasoning remains unclear. In our study, we verified the effect of working memory on Bayesian reasoning by evaluating the performance of participants with high and low working memory span (WMS); we also investigated if working memory as a kind of cognitive resource can affect Bayesian reasoning performance by manipulating the cognitive load in a dual-task paradigm among participants with no-, low-, and high-loads. We found the following: (1) The Bayesian reasoning performance of high WMS participants was significantly higher than that of low WMS participants. (2) Performance under natural frequency condition was noticeably higher than that in standard probability condition. (3) Interaction between working memory and probability format was significant, and the performance of participants with high-load in natural frequency condition was higher when compared to those of participants with no- and low-load. Therefore, we can conclude that: (1) Working memory resource is a major factor in Bayesian reasoning. The performance of Bayesian reasoning is influenced by working memory span and working memory load. (2) A Bayesian facilitation effect exists, and replacing the standard probability format with a natural frequency format can significantly improve Bayesian performance. (3) Bayesian facilitation occurs only in participants with sufficient working memory resources.

## Introduction

In daily life, people often make critical decisions based on conditional probabilities, such as in courts, hospitals and war rooms (Shi et al., 2019). Although decisions and judgments based on uncertainty are of great importance, reasoning performance based on probabilistic information is not satisfactory (Kahneman and Tversky, 1972). A good example of this is Bayesian reasoning. Bayesian reasoning is when people adjust their existing opinions based on new information or evidence to arrive at conclusions and make decisions. For example, the probability of breast cancer in the population is 1% for a woman who participates in routine screening. If a woman has breast cancer, the probability that she will have positive mammography is 80%. If a woman does not have breast cancer, the probability that she will also have positive mammography is 9.5%. If a woman in this group had positive mammography, what’s the probability that she has breast cancer? (Gigerenzer and Hoffrage, 1995).

Then we can calculate it as Bayes’ rules:

$$P\left(h|d\right)=\frac{P\left(h\right)P\left(d|h\right)}{P\left(h\right)P\left(d|h\right)+P\left(-h\right)P\left(d|-h\right)}$$

Where, P(h) represents the base rate of 1%, P(d| h) represents the hit rate of 80%, P(d|−h) represents the false alarm rate of 9.5%, and P(h| d) represents the posterior probability.

Since Edwards (1968) carried out his research on Bayesian reasoning, studies have consistently shown that people are not good decision-makers and their reasoning abilities on Bayesian problems are quite poor (Kahneman and Tversky, 1972; Gigerenzer and Hoffrage, 1995). However, Bayesian reasoning is common and deadly in People’s Daily life (Shi et al., 2019). Many researchers are trying to find ways to improve the reasoning performance. As for the influencing factors, previous studies mainly discussed the content (context) effect (Gavanski and Hui, 1992; Girotto and Gonzalez, 2002), the ways to obtaining probabilistic information (Lovett and Schunn, 1999) and factors of individual differences, such as knowledge background (Shi et al., 2006; Siegrist and Keller, 2011), cognitive style, cognitive responsiveness, numerical skills, emotional states and cognitive strategies (Sirota and Juanchich, 2011; Sirota et al., 2014; Reani et al., 2019).

Gigerenzer and Hoffrage (1995), Zhu and Gigerenzer (2006) found that when the natural frequency was used to represent probabilistic information, people and even children could perform Bayesian reasoning (Gigerenzer and Hoffrage, 1995; Zhu and Gigerenzer, 2006). This improvement has been confirmed by many studies (Cosmides and Tooby, 1996; Sloman et al., 2003; Barbey and Sloman, 2007; Sirota et al., 2014; Artur et al., 2015; McDowell and Jacobs, 2017; Weber et al., 2018).

There are two influential theories about the natural frequency promotion of Bayesian reasoning. One is the framework of ecological rationality, the other is nested set theory (Lesage et al., 2013). The ecologically rational framework argues that people perform better at natural numbers because people process natural frequencies better than probability. Some researchers found child couldn’t solve Bayesian problems when it was in probability format but they can solve Bayesian problems in natural frequencies. However, their performance was still not very high (Zhu and Gigerenzer, 2006) and for different individuals, the facilitation in natural frequency representation is not always working in every situation (Gigerenzer and Hoffrage, 1995; Cosmides and Tooby, 1996; Lovett and Schunn, 1999; Sloman et al., 2003; Barbey and Sloman, 2007; Miroslav and Marie, 2011). The nested set theory holds that under natural frequency conditions, the performance of the reasoning task could be promoted by making a collection of “nested” relationship visualization (De Neys and Schaeken, 2007; Barbet and Guillaume, 2016). However, many theories and research studies show that working memory plays a major role in solving reasoning problems (Johnson-Laird and Savary, 1999; Oberauer et al., 2005; Baddeley, 2007, 2010; Sejunaite et al., 2019). As the core of cognitive processing, working memory is closely related to the reasoning process, such as analogical reasoning and syllogism reasoning, and propositional reasoning (Meiser et al., 2001; Morrison et al., 2001; Markovits et al., 2002; Capon et al., 2003; Copeland and Radvansky, 2004; Sejunaite et al., 2019).

Their study showed that both working memory span and working memory resources were highly positively correlated with reasoning tasks (Meiser et al., 2001; Morrison et al., 2001; Markovits et al., 2002; Capon et al., 2003; Copeland and Radvansky, 2004). The theoretical model of the relationship between reasoning and working memory, such as the dual-process model, also holds that all kinds of cognitive processing activities are restricted by the working memory ability during reasoning (Johnson-Laird and Savary, 1999; Meiser et al., 2001). Bayesian reasoning is a type of probability reasoning, which originates from the process of making decisions and judgments based on the obtained information. The discussion of this problem can be active in improving the research field of working memory and reasoning.

Researchers have explored cognitive processing, such as cognitive reaction ability, to investigate the facilitation effect (Gigerenzer and Hoffrage, 1999; Süß et al., 2002; Miroslav and Marie, 2011; Lesage et al., 2013; Sirota et al., 2014). Even children could do Bayesian reasoning by natural frequency format representation (Zhu and Gigerenzer, 2006; Artur et al., 2015; McDowell and Jacobs, 2017). In other studies, researchers examined the relationship between Bayesian reasoning performance, cognitive reflective ability and individual development under different problem formats, and conducted experimental operations on cognitive resources under the dual-task paradigm. Results showed that the performance of Bayesian reasoning tasks depends on the participants’ general cognitive abilities (Lesage et al., 2013).

To further research the relationship between working memory and Bayesian reasoning, we expand upon the study of Lesage et al. (2013) in two aspects. To investigate the cognition process and rules of Bayesian reasoning and guide people in making effective decisions and judgments, we discuss both working memory and the probability format in this study. We assume that (1) working memory is closely related to Bayesian reasoning performance and that (2) there is a facilitation effect of natural frequency representation, but that the effect requires working memory resources. Two experiments were designed in this study. Experiment 1 is meant to study the influence of working memory span and probability format on Bayesian reasoning. The causal relationship of the working memory resource in the Bayesian inference task is not inferred from experiment 1 only. The dual-task can well study the central executive components involved in cognitive activities (Logie et al., 1994) and the introduction of an auxiliary task is an effective way to examine whether a process is dependent on the cognitive resource (De Neys, 2006; De Neys and Verschueren, 2006; De Neys and Schaeken, 2007; Khemlani et al., 2018; Kimura and Matsuura, 2019). Therefore, experiment 2 intends to design a dual-task experiment to further explore the mechanism of working memory in the reasoning process by giving a working memory load to manipulate the available working memory resource.

## Experiment 1: Effects of Working Memory Span on Bayesian Reasoning

### Purpose and Hypothesis

This experiment is aiming to investigate the effect of WMS on Bayesian reasoning and Bayesian facilitation. Based on previous studies, the hypotheses are as follows: (1) working memory span is highly correlated with Bayesian reasoning score, the performance of high WMS group was better than those of low WMS group; (2) there is a natural frequency facilitation effect.

### Methods

#### Participants

This experiment was approved by the Ethics Committee of Hunan Normal University, and written informed consent was obtained from all participants before the experiment was started. The sample size was calculated with a power of 0.8 and the minimum requirement of sample size was 36. 120 college students (male = 37, female = 83) participated in this experiment, with ages ranged from 18 to 25 years (mean ages = 20.5, SD = 3.45). All of the subjects volunteered to participate in the experiment and did not learn or understand Bayesian reasoning.

**FIGURE 1:**
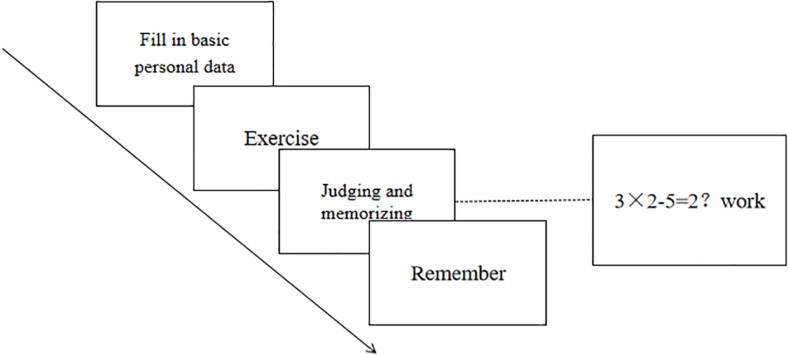
Flow chart of Operation Span Task.

This experiment used Operation Span Task designed by Turner and Engle (1989), and revised by Song et al. (2011) (see Figure 1). It’s made up of 75 mathematical equations with words chosen from the Dictionary of Modern Chinese Frequencies (1986 revised edition), double word noun, neutral. The word frequency is 0.0100–0.1429. The equations are all mixed operation of the multiplication (division) and the addition (subtraction), and the results are also in the single digits.

The WMS index was represented by the total number of double-character words correctly recalled in the group, with a range from 0 to 60. To ensure the validity of the subjects’ participation in the dual-task, the correct rate of the secondary task (equality judgment) was required to be more than 85% (Ikeda and Kitagami, 2013). In the end, the data from 48 subjects were eliminated because the experiment was interrupted or the correct rate of equality judgment was less than 85% and 72 subjects were selected and divided into high WMS and low WMS groups to participate in the formal experiment. The average and standard deviation for each group are shown in Table 1.

**TABLE 1:** Descriptive statistics of working memory span in high and low WMS groups.

Group	*N*	*M*	*SD*
High-WMS	36	54.47	3.19
Low-WMS	36	44.08	3.61

*In the independent sample t-test, t (70) = −12.93, p < 0.001, and d = 0.36. This indicates that the difference between the high and low WMS groups is significant and the classification is effective in this experiment.*

#### Materials and Instruments

A total of 5 of the 10 Bayesian problems (all problems are homogeneous) were extracted from materials developed by Zhu and Gigerenzer (2006) as the reasoning material. The problems were converted to an 800 × 600 pixel picture with black numbers on a white background. The text was in 21 Song style and 1.5 line spacing, and it was placed in the center of the picture. The procedure was run by E-prime 2.0 and rendered on a 19-inch DELL screen with a refresh frequency of 150 Hz and a resolution of 1024 × 768.

**FIGURE 2:**
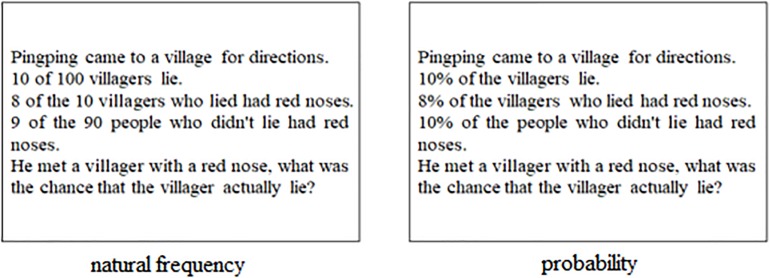
Frequency and possibility version of the same Bayesian reasoning problem.

Here are two versions of the same Bayesian reasoning question (the Red Nose problem) (see Figure 2):

#### Procedure

**FIGURE 3:**
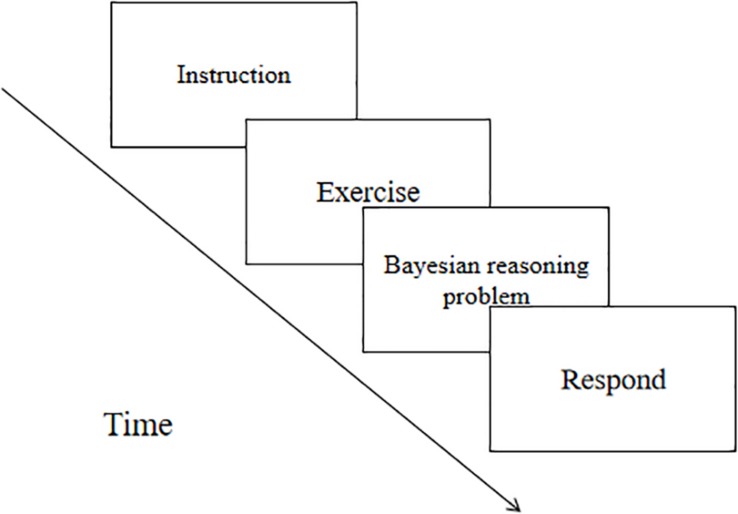
Flow chart of Experiment 1.

The procedure consisted of an exercise part and a test part. When the subjects familiarized themselves with the probabilistic question of the exercise part, they could start the second part of the formal experiment. The instructions were presented first and then entered the reasoning task. After the reasoning questions were presented, they analyzed and calculated on the paper and input the results into the answer box when they were completed (see Figure 3). At the end of the experiment, a small gift was given to the subjects.

### Results and Analysis

**FIGURE 4:**
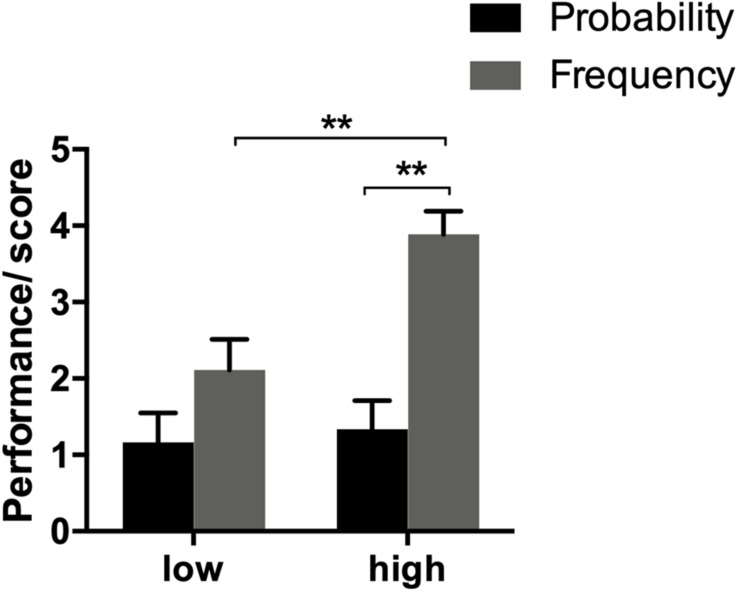
Results of different WMS subjects. ^∗^*p* < 0.05 and ^∗∗^*p* < 0.01.

The results of reasoning score of the 72 subjects were regarded as “correct” when the difference from standard answers was less than 1%; otherwise, they were regarded as “wrong.” Correct answer was scored as 1 while incorrect answer was scored as 0, and the total score was between 0 and 5 points. The SPSS19.0 software was used to analyze the data. The analysis and results are shown in Figure 4.

Two-factor ANOVA showed that the main effect of WMS was significant [*F*(1,68) = 6.967, *p* < 0.05, η^2^ = 0.10], that is, the Bayesian reasoning performance of high WMS subjects was significantly higher than that of low WMS subjects. The main effect of data format was significant [*F*(1,68) = 22.574, *p* < 0.05, η^2^ = 0.25], indicating that the results of reasoning by means of natural frequencies were significantly better than those of reasoning by means of standard probability. Interaction between WMS and data format was significant [*F*(1,68) = 4.783, *p* < 0.05, η^2^ = 0.07].

A further simple effect analysis showed the following:

At the high WMS level, the probability format effect was significant (*F* = 22.15, *p* < 0.05), meaning that the results of the high WMS subjects who used natural frequencies reasoning were significantly higher than the results of the subjects in the standard probability.

At the low WMS level, the probability format effect was not significant (*F* = 3.03, *p* > 0.05), that is to say, there was no significant difference between the results of low WMS subjects who used standard probability reasoning and the results of low WMS subjects who used natural frequencies.

The results showed that the working memory span is highly related to Bayesian reasoning, that the performance of the high WMS group was higher than low WMS, and the facilitation is more significant.

Although the results of experiment 1 showed that WMS is closely related to Bayesian reasoning, it was not sufficient for inferring the causality of working memory resources in the Bayesian reasoning task. Some studies have shown that the dual-task experiment can also explore the central executive components involved in cognitive activities and that the introduction of auxiliary tasks is also an effective way to examine whether a reasoning process depends on cognitive resources.

## Experiment 2: the Influence of Working Memory Load on Bayesian Reasoning

### Purpose and Hypothesis

Based on the dual-task paradigm (Logie et al., 1994; Khemlani et al., 2018; Kimura and Matsuura, 2019), the mechanism of working memory in the reasoning process is further explored by directly placing a load on the individual’s working memory (De Neys and Verschueren, 2006; De Neys and Schaeken, 2007). The experimental assumptions are as follows: (1) working memory load affects the reasoning performance of subjects; and (2) there is a Bayesian facilitation effect, but it does not exist in all load conditions.

### Methods

#### Participants

This experiment was approved by the Ethics Committee of Hunan Normal University in China, and written informed consent was obtained from all participants prior to the experiment. A necessary sample size of 42 was calculated by G-Power 3.1 with power = 0.8 (Iachini et al., 2005). 136 paid college students (male = 42, female = 96) aged 18–25 years (mean ages = 23.3, SD = 3.24) participated in this experiment.

### Materials and Instruments

The secondary task designed by Si et al. (2012) was used as an alphabetical order task (high-load) and a letter recognition task (low-load). The main task selected two of 10 Bayesian problems (all of which are homogeneous) from Zhu and Gigerenzer (2006) (different from the five reasoning questions in experiment 1). The reasoning questions were converted to 800 × 600 pixel pictures with black characters on a white background. The characters were presented in 21 Song style (34 letters), 1.5 line spacing and the text was in the center of the picture. Programmed and run by E-Prime 2.0, the picture was presented on a 19-inch dell computer screen with a display refresh frequency of 150 Hz. The resolution was 1024 × 768. The same reasoning question was presented in two versions: standard probability and natural frequency.

#### Design

**FIGURE 5:**
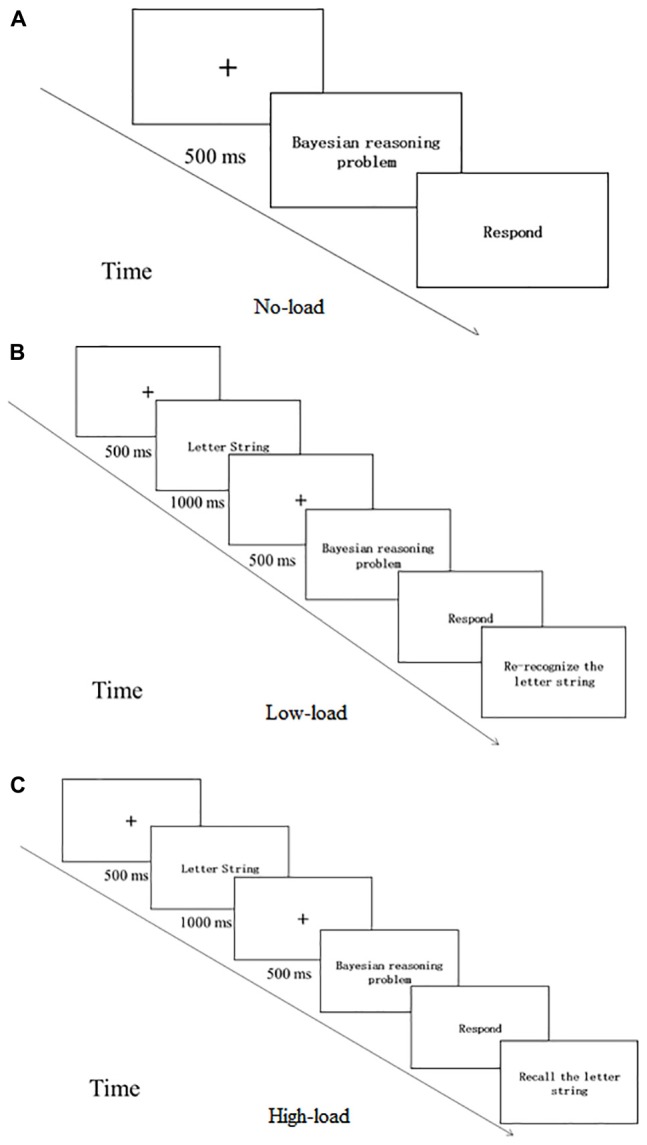
Flow chart of Experiment 2. **(A)** is for No-load condition, **(B)** is for the Low-load condition and **(C)** is for the High-load condition.

The subjects completed Bayesian reasoning problems in probability format and natural frequency format. The working memory resources were manipulated by the secondary task of the dual-task paradigm. The factor working memory load distinguished between the high-load, low-load and the control condition. In the dual-task condition, participants were presented with a letter string (i.e., AGRCWO) which they were instructed to keep in mind while solving the Bayesian reasoning problems. Subjects in the high-load condition need to recall the letter string while in the low-load condition only need to re-recognize the letter string (Si et al., 2012). No letter string was presented in the no-load condition (see Figure 5). The dependent variables were the subjects’ score on 2 Bayesian reasoning questions (1 for a correct answer, 0 for an incorrect answer, the total score was 0∼2).

### Procedure

### Results and Analysis

Based on the finding of experiment 1, to further explore the working memory and if the working memory resources affect the Bayesian reasoning. Four subjects did not complete the reasoning questions or interrupted the experiment. The average score of the two alphabetical tasks (1 for a letter) and the two-letter recognition tasks (the correct answer score was 1 point while the incorrect answer was 0 points) were calculated and the data from eight subjects were excluded because their score of the secondary task was below the average three standard deviations. And the data of the remaining 124 subjects can be retained.

**FIGURE 6:**
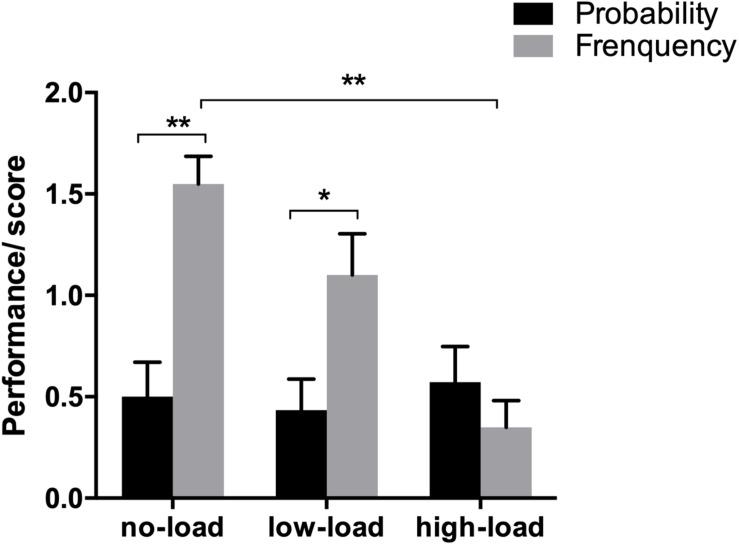
Results of different WM loads and data formats. ^∗^*p* < 0.05 and ^∗∗^*p* < 0.01.

If the difference between the results of the reasoning and the standard answer was less than 1%, the answer was regarded as “correct,” whereas regarded as “wrong,” a correct answer scored as 1, and the total score was 0∼2. The results are shown in Figure 6.

Two-factor ANOVA showed that the main effect of the working memory load was significant [*F*(2,118) = 5.861, *p* < 0.05, η^2^ = 0.09]. The results of reasoning in the three working memory load conditions were as follows: the best results were in the no-load condition, the second was in the low-load condition, and the worst was in the high-load condition. The main effect of the data format was significant [*F*(1,118) = 13.896, *p* < 0.05, η^2^ = 0.11]. The reasoning results of subjects in the natural frequency scenario were significantly better than those of subjects who were in the standard probability scenario. Interaction between working memory load and data format was significant [*F*(2,118) = 7.838, *p* < 0.05, η^2^ = 0.12].

A further simple effect analysis showed the following:

At the no-load level, the data format effect was significant (*F* = 18.49, *p* < 0.05), which showed that the reasoning results of the subjects in the natural frequency scenario were significantly better than those of subjects in the standard probability scenario.

At the low-load level, the data format effect was significant (*F* = 7.90, *p* < 0.05), that is, at the low working memory load level, the reasoning score of the subjects in the natural frequency scenario were significantly better than those of subjects in the standard probability scenario.

At the high-load level, the data format effect was not significant (*F* = 0.74, *p* > 0.05). At the high-load level, there was no significant difference in reasoning performance between the two data formats.

## General Discussion

### Impact of Working Memory on Bayesian Reasoning Performance

The results of experiment 1 showed that the performance of Bayesian reasoning was influenced by the working memory span (WMS). This is consistent with the researches of Capon et al. (2003), Copeland and Radvansky (2004), and Bai et al. (2004) of other types of reasoning.

Bayesian reasoning is a kind of complicated probabilistic reasoning that involves a series of probability information and rules. Individuals with high WMS have more ability and resources to use probability rules, so they could integrate all kinds of explicit information relationships and operate on numbers, and can result in the answer smoothly. However, for individuals with low WMS, it is impossible to integrate and calculate the probability information effectively because the complex cognitive process is far beyond their own WM ability. Therefore, the reason why individuals cannot obtain the correct answer when completing the experimental task is that they guess or input an answer randomly according to their own intuitive judgments, which lead to a reasoning error (Lesage et al., 2013).

The results of experiment 2 showed that WM load can affect Bayesian reasoning. Specifically, the degree of influence increases with WM load. The reasoning performance was best in the no-load condition, second in the low-load condition and the worst in the high-load condition.

For each individual, the WM resource remains relatively stable and limited. When a secondary task occupies more WM resources, they have fewer resources to solve the main task (Robbie et al., 1982). Of the three conditions, the alphabetical recall was the one that takes up most of the WM resources; thus, under a condition of high-load, subjects had the least resources to address the Bayesian reasoning problem, so this result was the worst. The task of alphabetical recognition was simpler than the task of alphabetical sorting and takes up fewer resources. Therefore, there were more WM resources used to solve the problem, thus the performance under low-load condition was significantly improved. In the no-load condition, there were the only tasks competing for the limited WM resources, so the subjects showed the best performance.

### Bayesian Facilitation Effects

Sloman et al. (2003) called the improvement of Bayesian reasoning performance under natural frequency conditions Bayesian facilitation. Both experiments 1 and 2 showed an obvious Bayesian facilitation effect: the subjects’ results in natural frequency scenario were significantly higher than those in the standard probability scenario. This was consistent with the results of Gigerenzer and Hoffrage (1995), Cosmides and Tooby (1996), Brase et al. (1998), Artur et al. (2015), and McDowell and Jacobs (2017).

Therefore, two experiments verified the Bayesian facilitation in natural frequency representation. However, the Bayesian facilitation effect did not appear under all conditions, that is, the occurrence of the Bayesian facilitation effect requires certain conditions. The results of experiment 2 verified the facilitation effect. The results showed that the performance in natural frequency format was significantly better than in the standard probability format under low-load and no-load conditions, but the difference between them was not significant under a high-load condition. This indicated that the Bayesian facilitation effect was only reflected in the low-load and no-load conditions, but it was not found in the high-load condition.

Working memory resource is a kind of important cognitive resource for reasoning (Meiser et al., 2001; Morrison et al., 2001; Markovits et al., 2002; Capon et al., 2003; Copeland and Radvansky, 2004; Baddeley, 2007, 2010; Sejunaite et al., 2019). Under high-load conditions, secondary tasks (alphabetical recall) occupied more WM resources, and few WM resources are used to complete the main task. As a result, the subjects in the two versions of the reasoning problem had poor scores. However, in the low-load or no-load conditions, the results were the opposite. Most of the WM resources were utilized for processing reasoning tasks. Although they performed poorly on reasoning problems with the standard probability format, they were able to accomplish relatively simple reasoning problems presented in natural frequency format (Johnson-Laird and Savary, 1999; Lesage et al., 2013).

### The Explanation for the Effect of Working Memory on the Bayesian Facilitation

Experiment 1 found that under the natural frequency condition, the score of the high WMS group was significantly higher than those of low WMS. Experiment 2 also found that under the natural frequency condition, the reasoning results of subjects in low-load and no-load conditions were significantly higher than those under the condition of probability (Miroslav and Marie, 2011; Lesage et al., 2013; Sirota et al., 2014).

The ecologically rational framework argues that people perform better at natural numbers because people process natural frequencies better than probability. And the computational requirements for natural frequencies are much simpler than for probabilities. However, the participants couldn’t complete the problems well in high-load conditions. The nested sets theory holds that there is a positive relationship between working memory resources and reasoning performance, especially under the natural frequency condition that nest-set is clear (De Neys and Schaeken, 2007; Barbet and Guillaume, 2016). The results of experiment 1 showed that the working memory span was highly related to the inference performance. Moreover, the reasoning score of individuals with a high working memory span is significantly better than those with low working memory span under the natural frequency condition. The results of experiment 2 also show that the result of reasoning depends on available working memory resources and the same as the facilitation under natural frequency condition.

The nested sets theory is proposed on the basis of the dual-process model, which makes the structure of the problem set clear and triggers the analysis system. The system uses executive cognitive resources to calculate the correct answer. Therefore, the reason why people perform better with natural frequency is that the nest-set is clear by making a collection of “nested” relationship (the larger subset embedded collection) visualization (Shi et al., 2006; Barbey and Sloman, 2007). That is, under the condition of arousing a clear nested sets representation, the reasoning performance of the subjects should be related to the general cognitive ability of the individual: the more cognitive resources there are the more likely it is that the individual obtains the correct answer. In contrast, under the condition of a fuzzy representation of the problem, they are unable to successfully complete reasoning tasks (De Neys and Schaeken, 2007; Artur et al., 2015; Barbet and Guillaume, 2016).

The results support the effect of working memory on reasoning. If the cognitive process is too complex that it exceeded people’s working memory ability, their reasoning will be wrong. Bayesian reasoning is a very difficult probability reasoning that involves a series of probability rules such as addition, multiplication, division, and a complex cognitive process. The use and storage of the information, rules, and the calculation of the premise and new information are all restricted by working memory capacity (Johnson-Laird and Savary, 1999; Meiser et al., 2001; Khemlani et al., 2018; Kimura and Matsuura, 2019). For individuals with high working memory resources, because they have a strong ability to calculate numbers, use probability rules and integrate all kinds of explicit information relations, so they could calculate the answers more smoothly. But for individuals with low working memory resources, this complex cognitive process far exceeds their working memory ability, so they cannot effectively integrate and calculate the probability information and draw the correct conclusion. This means, that they only complete the task of random speculation or according to their own intuitive judgment arbitrary to input an answer, resulting in reasoning errors (Capon et al., 2003; Copeland and Radvansky, 2004; Khemlani et al., 2018).

## Conclusion

Working memory resource is an important factor that influences Bayesian reasoning performance; that is, the quality of Bayesian reasoning results depends on working memory resources. Individuals with high WMS or sufficient resources exhibit better cognitive processing than those with low WMS or insufficient resources. However, this advantage is not always true, and it may not exist when the cognitive task is too hard. A Bayesian facilitation effect exists and replacing standard probabilities with natural frequency can greatly improve Bayesian performance. However, only in individuals with high working memory span or sufficient cognitive resources does this effect occur. The experimental results provide experimental evidence for the effect of working memory on Bayesian reasoning.

## Data Availability Statement

The original contributions presented in the study are publicly available. This data can be found here: https://figshare.com/articles/data/11827053.

## Ethics Statement

This experiment was approved by the Ethics Committee of Hunan Normal University in China, and written informed consent was obtained from all participants prior to the experiment.

## Author Contributions

ZS, LY, and ZL conceived and designed the study. LY and ZL performed the experiments and wrote the manuscript. LY, ZL, and SP analyzed the data. ZS, LY, TT, and YX the manuscript. All authors contributed to manuscript revision, read, and approved the submitted version.

## Conflict of Interest

The authors declare that the research was conducted in the absence of any commercial or financial relationships that could be construed as a potential conflict of interest.
